# Integrating theory and practice: the core components guide for rigorous quality improvement design

**DOI:** 10.3389/frhs.2026.1751580

**Published:** 2026-03-25

**Authors:** Paul Howard, Jafet Arrieta, Chelsey Leruth, Kate Bones, Rebecca Steinfield, Johanna Figueroa, Sonya Panjwani Olaya, Pierre Barker

**Affiliations:** 1 Institute for Healthcare Improvement, Boston, MA, United States; 2 Department of Global Health and Social Medicine, Harvard Medical School, Boston, MA, United States; 3 Harvard TH Chan School of Public Health, Boston, MA, United States

**Keywords:** core components, healthcare, implementation science, improvement science, QI design, quality improvement, quality improvement evaluation

## Abstract

Quality improvement (QI) methods have been used extensively to support the delivery of safe, timely, effective, equitable, and cost-effective health care. While QI initiatives have demonstrated benefits, critical gaps in design and implementation undermine their impact. Systemic reviews and expert commentaries point to recurring challenges, including limited understanding and appreciation of the system in which the work takes place; poorly articulated aims; absence of guiding content theories for scalable implementation; weak implementation strategies; inadequate mechanisms for measurement, evaluation, and learning; and insufficiently structured approaches to communication and dissemination. These gaps limit learning, impact, replication, sustainability and scalability. To address these gaps, the Institute for Healthcare Improvement (IHI) developed the Core Components Guide, a practical framework for designing, implementing, and evaluating QI initiatives. Grounded in Deming's System of Profound Knowledge and the Model for Improvement, the guide includes six interrelated components: System Understanding, Improvement Aim, Measurement, Evaluation and Learning, Content Theory, Execution Theory, and Dissemination and Communication. Together, these components provide a structured approach to align interventions with context, clarify program theory, and embed iterative learning cycles. This manuscript introduces the Core Components, illustrates their application through a case study, and shares lessons learned from operationalizing the guide across diverse settings. By integrating improvement and implementation science principles, the Core Components Guide strengthens design, promotes fidelity, and increases the potential for impact, replication, scale, and sustainability of QI initiatives. This Guide offers actionable strategies for QI leaders and policymakers to build stronger foundations for improvement, evaluation, and dissemination.

## 1 Introduction

Quality improvement (QI) methods have been utilized in health and health care settings to support the delivery of safe, timely, effective, efficient, equitable, and cost-effective care (1–3). The use of QI has evolved from isolated local projects to holistic deployment across complex systems (4). Previous research shows that applying QI can lead to safer care, better outcomes, and lower costs (5, 6), and can empower providers to deliver both person-centered and context-specific care; boosting engagement, teamwork, and job satisfaction (7).

However, gaps in the design and implementation of QI initiatives often undermine their effectiveness (8–10). First, inadequate system appreciation remains a challenge. Without clearly defining the problem, the system, and the surrounding context, improvement teams may overlook important influences and fail to identify factors contributing to the problem (11). Second, QI initiatives often have poorly defined aims. Aims that are not specific, measurable and time-bound create ambiguity and limit evaluation efforts (8). Third, the absence of a content theory limits the adoption, adaptation, and reliable implementation of evidence-based interventions (12). Fourth, approaches to guide improvement teams in testing and implementation can be vague, overly ambitious, or disconnected from operational realities (8, 13). Similarly, the absence of adaptive learning principles, such as Plan-Do-Study-Act (PDSA) cycles, can contribute to the failure of QI initiatives (8, 14). Fifth, failure to design and embed measurement, evaluation, and learning (MEL) mechanisms throughout the project lifecycle limits the ability to assess progress and establish a causal pathway (10). Finally, the lack of structured communication and dissemination strategies can limit the spread and scale of successful interventions.

Recent systematic reviews and commentaries have highlighted these challenges, emphasizing the need for greater integration of improvement and implementation science and approaches to inform the design and implementation of QI initiatives (9, 10, 15). Given the variable results of QI initiatives, the field could benefit from specific guidance to designing QI initiatives with a clear program theory that is more likely to lead to learning, improvement and impact (16, 17).

This manuscript introduces the Core Components, a practical guide developed by the Institute for Healthcare Improvement (IHI) to address these challenges. The Core Components Guide offers an integrated approach to improvement in complex systems, drawing on improvement science, implementation science, evaluation, and related fields. Together, they provide a practical yet robust structure to guide the design, implementation, evaluation, and dissemination of QI initiatives, while helping practitioners integrate knowledge generation, research, and practice. Their use creates synergies across these domains by:

- Grounding changes to be tested in shared theory and evidence (knowledge)
- Generating evidence about changes that result in improvement that can be used by others and/or to bring improvement to scale (research)
- Embedding all activities and learning within real-world settings “in context” based on an understanding of the system(s) of focus (practice).

This approach also enables measurement and evaluation insights to inform both improvement and research, for research findings to lead to better practice, and for accumulated knowledge to accelerate improvement at a larger scale. The authors provide an overview of the Guide, illustrate its application through a case study, and discuss lessons learned in operationalizing it.

## 2 The core components guide

### 2.1 Origin of the core components

Informed by an IHI innovation cycle and decades of experience leading improvement initiatives, IHI developed its Core Components Guide in 2014 (18). The initial guide included five Core Components (Aim; MEL; Content Theory; Execution Theory; and Dissemination and Communication) and emphasized the value of formative, theory-driven evaluation that addressed a central learning question: “How and in what contexts does a new model work or can be amended to work?” (19). Recognizing the need to strengthen design and set the stage for evaluation, IHI expanded the use of the Core Components Guide to design, implement, refine, and scale-up improvement initiatives (20). In 2024, IHI added a core component of System Understanding to reinforce context-alignment in the design phase.

### 2.2 Underpinning frameworks

Two frameworks underpin the Core Components Guide: W. Edwards Deming's System of Profound Knowledge (SoPK) (21) and the Model for Improvement (MFI) (8). SoPK offers four interrelated domains to guide the design and implementation of improvement initiatives: appreciation for a system, knowledge of variation, theory of knowledge, and psychology (21). Anchored in SoPK, the MFI includes three questions: what are we trying to accomplish, how will we know that a change is an improvement, and what changes can we make that will result in improvement (22). These three questions drive adaptive testing and learning using PDSA cycles.

### 2.3 Overview of the core components guide

**Figure 1:**
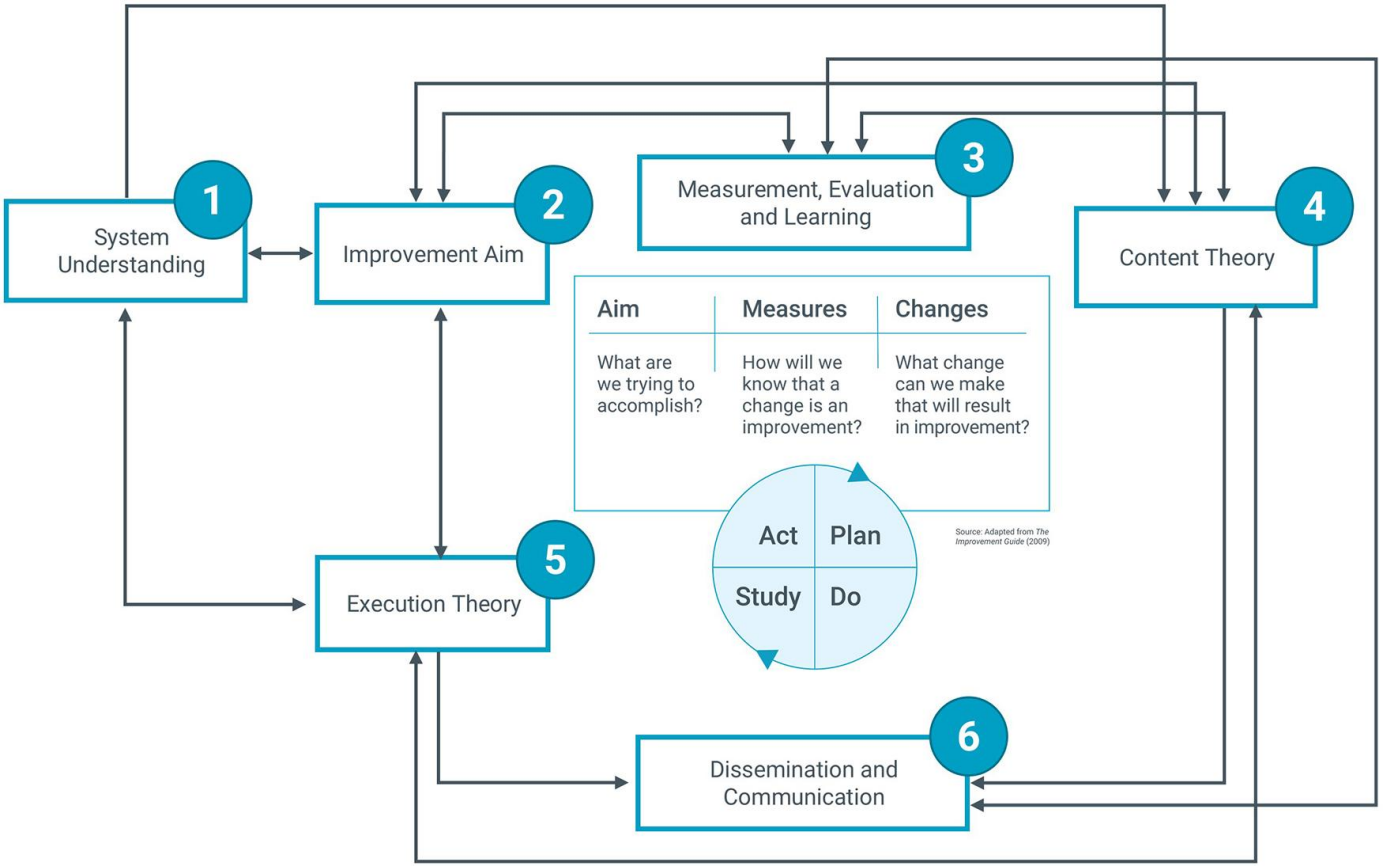
Core components guide mapped against the model for improvement (8).

The guide includes six interrelated Core Components (see Figure 1): (1) System Understanding; (2) Improvement Aim; (3) MEL; (4) Content Theory; (5) Execution Theory; (5) and (6) Dissemination and Communication. Like the MFI, the Core Components are agnostic to the aim, scope and scale of the QI initiative, type of organization, or context. The Core Components Guide encourages planning based on theory, prediction, evidence, and iterative learning.

#### 2.3.1 System understanding

“Appreciation of the system” is a required element for improvement in Deming's SoPK (21). Understanding how the system works—the different parts and how they interconnect and work together—is a critical starting point for integrating improvement into the unique context [system(s) or setting(s)]. It makes explicit the need to better understand the actors within the system, how things are currently done in the system(s) of focus, the competing priorities and motivations of participating teams, these teams’ bandwidth, the level of will among their leaders and staff and baseline performance. This work provides a “bird's eye” view to help improvement teams zoom out from isolated problems to understand larger root contributors to the system's function. Acknowledgement and exploration of power dynamics, structural inequities, and historical and current mistrust are also important to this component, as they influence how the system functions and for whom they improve outcomes (23–25).

System understanding starts during the initial design process, informs the development of the other Core Components and continues throughout the lifecycle of the initiative as changes are tested and refined for successful implementation. Five recommended activities help build system understanding:

**Problem Statement and Diagnosis**—an accurately understood problem and well-articulated problem statement.
**Context Assessment**—an analysis of the influencers of the project's success including factors related to the environment, social conditions, culture, etc.
**Evidence Review—**a review of formal and informal evidence to understand the broader landscape related to the work of the project.
**Data Review**—analysis of the system's data on past and current performance, variation in subsystems, and populations disproportionately impacted. This could include an analysis of needs, opportunities, and ways in which systems have discarded or undervalued assets of individuals and communities to identify ways these can be addressed to advance population health and dismantle inequities (26).
**Actor Engagement—**identification of the actors in the system (e.g., health care workers, patients, and families) to support future engagement in the project.

#### 2.3.2 Improvement Aim

Building off “system understanding,” an effective aim provides a shared vision for improvement and helps answer the first question of the MFI (8, 27). An aim statement should be specific, numeric, and time-bound and describe the gap between current performance and performance that is desired for key outcomes that matter to the people engaged in and impacted by the work. It should be ambitious yet feasible, reflecting an understanding of the system. A QI initiative may have an aim statement for the whole project (e.g., decrease average Hemoglobin A1C for the outpatient Type 2 diabetes population from 8.5% to 6.5% over 18 months) or a set of sub-aims relating to targeted process in the system of care (e.g., improve reliability of annual screening of Hemoglobin A1C for all returning Type 2 diabetes population from 60% to 95% in 6 months). For QI initiatives involving multiple teams, individual improvement teams are encouraged to adapt the initiative aim to their own context informed by their local baseline.

#### 2.3.3 Measurement, evaluation, and learning

Continuous MEL should inform all aspects of an improvement initiative. Most QI initiatives require modifications during their implementation to meet their stated aim(s), and robust, yet practical MEL activities provide the information needed to make informed decisions. The MEL Core Component guides improvement at two levels: (1) how the system of interest is changing (informed by the measurement strategy) and (2) the effectiveness of the QI initiative's implementation (informed by the MEL plan). While these areas of learning are interrelated, they have different primary users and purposes and utilize distinct methods and tools.

**Measurement Strategy**—helps teams implementing the QI initiative “on the ground” answer the second question of the MFI—whether the changes are leading to improvement (28). A robust measurement strategy includes a family of measures (outcome, process, and balancing measures) (28); clear operational definitions, a feasible plan for data collection across teams; and a system for visualizing and analyzing data over time, such as run charts or statistical process control charts (28–30). Assessing these measures over time enables teams to assess whether changes are resulting in improvement, adapt as needed, and make decisions.
**MEL Plan**—includes questions, data, and tools to inform the project team (individuals responsible for the overall design) and key actors about the QI initiative's progress, effectiveness, and learnings. Formative evaluation during the project facilitates adaptive design (31, 32), while summative evaluation at the conclusion of the project provides evidence about the causal pathway (20, 33).

A learning plan may include regular, formal, and informal activities to pause and reflect on what is working and what is not, and to use these insights to adapt the execution theory and content theory.

#### 2.3.4 Content theory

The content theory outlines the QI initiative's theory of change and includes the evidence-based interventions that are known to be effective in improving results and the system drivers to be acted upon to achieve the aim (34). The content theory constantly evolves, based on learning about which ideas and drivers work, and which do not.

Two tools can depict and organize the content theory:

**Driver Diagram**—a visual tool that articulates the high-level theory of change predicted to influence the achievement of the aim (34). Driver diagrams include the aim statement, primary drivers, secondary drivers, and change ideas. Primary drivers are key leverage points for improving the performance of the system and secondary drivers represent the factors, steps, processes or elements in the system that influence primary drivers. For each secondary driver, a set of change ideas is proposed (34, 35).
**Change Package**—a set of specific and actionable changes, often organized in alignment with the driver diagram, that may be tested, refined, and embedded into everyday practice by the improvement teams (36).

#### 2.3.5 Execution theory

The execution theory specifies *how* change will occur—detailing the mechanisms and implementation strategies that will drive reliable implementation of the content theory to achieve the intended aim. Developing a clear execution theory during the design phase of a QI initiative is critical to ensure that the other Core Components, particularly the content theory and measurement strategy, are implemented with fidelity and in a way that achieves the initiative's aim.

##### 2.3.5.1 Organizing the Execution Theory

A well-defined execution theory clarifies the necessary structures, inputs, activities, outputs, and implementation supports needed to improve outcomes (37). Inputs include the elements required to enable improvement to operationalize the content theory, such as leadership commitment, protected time for staff participation, data systems, and an improvement model (e.g., the Model for Improvement or Lean). Activities refer to the processes that promote learning and collaboration, such as structured learning sessions, coaching, site visits, peer-to-peer exchanges, and dashboards that support real-time data analysis and feedback. Outputs include tangible products such as the number of PDSA cycles completed, percentage of teams submitting data, or percentage of teams receiving coaching (38, 39).

Two helpful tools to display the execution theory are a project roadmap and a logic model.

**Project Roadmap—**specifies the project's key phases, activities to be delivered, and milestones required to translate plans into reliable and effective implementation based on the selected change model. Sample activities detailed in the roadmap are in-person or virtual learning sessions, coaching calls, site visits, etc. The roadmap clarifies the sequence and dosing of the activities predicted to facilitate engagement, build QI capability, and support peer learning.
**Logic Model**—a visual representation of how the initiative works to achieve its aim. A logic model displays relationships between the inputs required to implement the project, activities, outputs, and short-, medium- and long-term outcomes expected as a result (14, 40, 41). The logic model is a useful way to graphically assemble the Core Components into a theory.

These tools should define roles and accountability structures, data systems for data collection and visualization, timely feedback mechanisms for communication and learning, and ongoing capacity building (37, 42). Integrating explicit execution planning helps strengthen implementation fidelity, anticipate and address challenges, align resources, promote adaptive learning, and increases the likelihood of achieving measurable and sustained improvement (37).

#### 2.3.6 Dissemination and communication

Effective dissemination and communication of results and insights from QI initiatives are essential to maximize their impact, promote shared learning, and accelerate system-wide change. A clear dissemination and communication plan helps ensure that learning does not remain local but instead informs broader improvement efforts (43, 44). Effective dissemination increases transparency, strengthens credibility, and contributes to the science of improvement by enabling replication, adaptation, and evidence-informed decision-making (45). It also helps build will for change and ensures that insights about what works—and what does not—reach diverse audiences, including frontline providers, leaders, policymakers, and communities (46–48).

A dissemination and communication plan should consider what project learning might be of interest to others (internally and externally), who the potential audience(s) are, and what platforms and outputs are useful for reaching them. The plan should specify the resources (people, time, etc.) required to execute these activities, including staff expertise, data visualization, and publication fees (47, 48). Internal dissemination products include peer-learning events, pause and reflect sessions, and internal knowledge bases. External dissemination products include peer-reviewed publications, conference presentations, knowledge management tools, blogs, videos, storyboards, how-to-guides, toolkits, and project summaries.

## 3 Case study: global comfort promise

Global Comfort Promise, a QI initiative implemented by IHI and St. Jude Children's Research Hospital, developed and refined the Core Components to guide the effective delivery of an evidence-based intervention across multiple sites globally. The content theory and measurement strategy were developed using a series of expert panels with subject matter experts and people with lived experience over four months. A detailed case study is available in Supplementary File S4.

## 4 Discussion

QI has demonstrated potential to improve safety, outcomes, and efficiency across health systems. However, gaps in design and implementation of QI initiatives hinder their effectiveness (19, 49) and limit learning, impact, replication, and scalability (50, 51). The Core Components Guide offers a structured approach to address these challenges and includes actionable strategies for QI leaders, project teams, evaluators, researchers, and policymakers to strengthen the design, implementation, and evaluation of QI initiatives. The Core Components Guide is not intended to replace established QI, implementation science, or evaluation frameworks; rather, they integrate and operationalize these into a coherent, practice-ready structure that connects design, implementation, and evaluation throughout an improvement effort.

While the Core Components can strengthen any QI initiative, they are particularly valuable for efforts that require accountability for outcomes, are designed for scale, or involve complex, multi-site or multi-partner implementation. IHI has applied the Core Components to guide the design of complex QI initiatives since 2014. Between January 2024 and September 2025, 21 of 23 IHI project teams across six continents used the Core Components Guide, providing a standard approach to project design, implementation, learning, and evaluation.

The Core Components are interdependent elements within the design of a QI initiative; each one is a critical element in the blueprint for how the initiative should be implemented. As such, design typically follows a non-linear, iterative process. However, system understanding stands apart as a foundational element that underpins the other Core Components; it plays a critical role in shaping the initiative's aim, content theory, and measurement strategy. For this reason, designers must invest early in deeply understanding the system at hand. The remaining components should then be developed in alignment with that understanding and with one another, ensuring that interventions are context-sensitive and aligned with organizational priorities (see Table 1) (11, 52, 53).

**Table 1:** The 6 core components for designing quality improvement initiatives.

Core component	Questions the core component answers	Activities
System Understanding	How do we understand the system(s) we seek to improve in all its complexity, including past and current performance in the outcomes that matter? What are the known problems and contributors to those problems? Who are the key people involved in the system and affected by the outcomes?	Define and diagnose the problem Assess context Review evidence Review data Identify & engage actors
Improvement Aim	What are we trying to accomplish? How much improvement? For whom? By when?	Determine project aim
Measurement, Evaluation, and Learning	What measures will guide improvement teams to success? What additional data, methods, and tools will help the project team evaluate if, how, and why the project is working? How can we build or strengthen the learning systems?	Develop measurement strategy Design evaluation and learning plan
Content Theory	What are the demonstrated solutions that should inform our work? What evidence-based ideas, innovations and system changes are most likely to drive us towards our aims?	Develop driver diagram Identify change Ideas/develop change package
Execution Theory	What resources and planned activities will enable us to achieve the desired outcomes? What is the theory for how the project inputs and activities will produce the expected outputs and outcomes?	Select (and adapt as needed) design (e.g., Breakthrough Series Collaborative) Develop logic model Develop roadmap
Dissemination and Communication	How will we capture and share what we are learning and the impact we are making? To whom? How often? Through which channels?	Develop dissemination plan including: Communications strategy Opportunities for publications and presentations Resources for scale

The Core Components should be co-designed with subject-matter experts, people with lived experience, and those directly involved in the system(s) of focus—they cannot be created in isolation.

The guide also emphasizes adaptive design: the Components should function as a living document, continuously refined as new learning emerges. Embedding iterative testing through PDSA cycles strengthens adoption, implementation fidelity, and ongoing learning and adaptation (54).

The Core Components are not a panacea. Things can (and do) go wrong when implementing QI initiatives. However, by laying a strong foundation for design, the Core Components set the stage for effective implementation. Similarly, planning for evaluation, learning, and dissemination activities enables meaningful learning and sharing during and after a QI initiative. Clarity about the project aim, content theory, and execution theory make the project evaluable; improvers cannot determine if, how, and why the project is effective if they have not made explicit the aim and underlying theories (55).

## 5 Limitations and future directions

Effectively operationalizing the Guide requires capability building, leadership engagement, and robust data infrastructure (19, 37, 56). This Guide draws on the improvement science, implementation, and evaluation literature, as well as iterative learning from IHI projects across six continents. Although not formally validated, its grounding in real-world practice enhances its relevance. Further research using common measures is needed to validate the Guide's usefulness across contexts and assess if adherence to the Core Components improves design quality, implementation fidelity, outcomes, and learning for QI projects.

## Data Availability

The original contributions presented in the study are included in the article/Supplementary Material, further inquiries can be directed to the corresponding author.
