# Supplementary material for: Integrating theory and practice: the core components guide for rigorous quality improvement design
Source: Front Health Serv. 2026 Mar 25;6:1751580. doi: 10.3389/frhs.2026.1751580 (PMC13057544; doi:10.3389/frhs.2026.1751580)
Supplement: Supplementary file 1 [file Table1.docx]

**Supplemental File 1: Deming’s System of Profound Knowledge and the 6 Core Components**

Deming’s System of Profound Knowledge offers four interrelated domains to guide the design and implementation of improvement initiatives: appreciation for a system, knowledge of variation, theory of knowledge, and psychology (1). The following table provides a brief overview of these domains and describes how they connect to the 6 Core Components.

| **System of Profound Knowledge Domain** | **How Domain Relates to Core Components** |
| --- | --- |
| **Appreciation for a system** - involves a deep understanding of how the different parts of an organization interact and how they might contribute to the problem to be addressed. | This domain relates most closely with the first Core Component – **System Understanding**, and informs the five recommended activities: problem statement and diagnosis, context assessment, evidence review, data review and actor engagement. |
| **Understanding variation** - requires being able to distinguish between common cause (random) and special cause (non-random) variation to assess progress over time and to guide improvement and decision-making (1). | This domain relates to:   - **System Understanding**, specifically the activities related to Data Review – analysis of the system’s past and current performance, variation in subsystems, and populations disproportionately impacted by the problem. - **Measurement, Evaluation and Learning**, including a system for visualizing and analyzing data over time, such as run charts or statistical process control (SPC) charts. |
| **Theory of knowledge** - being grounded in explicit theories—predictions about what changes will lead to improvement—and the importance of testing these predictions through iterative and adaptive learning using methods such as PDSA cycles (1,2) | This domain relates most closely to **Content Theory**, the evidence-based interventions that are known to be effective in improving results and the system drivers (primary and secondary) that need to be acted upon to achieve the aim.  In addition, the **Measurement, Evaluation, and Learning** component provides the measurement strategy and resulting “evidence” by which degree of belief in the content theory can be increased (3). |
| **Psychology** - addresses the human dimension of change. Deming recognized that improvement requires actively engaging people, promoting collaboration, and addressing resistance to change (1,4). | This domain relates to:   - **Systems Understanding**, including activities related to context assessment and actor engagement - **Content Theory**, including the system drivers (primary and secondary) that need to be acted upon to achieve the aim, and - **Execution Theory**, including designs that include efforts to develop the “will” needed for change and methods that promote group learning such as storyboard rounds and all-teams calls. - **Measurement, Evaluation, and Learning**, specifically the inclusion of qualitative methods that in the evaluation plan in order to give voice to and contextualize the experience of people involved in and affected by the initiative as well as the design or strengthening of learning systems that provide opportunities for interpretation of results with actors across the system. - **Dissemination** **and Communication**, including both internal and external knowledge sharing. In particular, storytelling is a powerful vehicle for change that activates people’s intrinsic motivation and has demonstrated potential for improving the impact of QI initiatives (5). |

**References**

1. Deming W. The New Economics for Industry, Government, Education. MIT Press; 1994.

2. What’s Your Theory? | ASQ [Internet]. [cited 2025 Aug 26]. Available from: https://asq.org/quality-progress/articles/whats-your-theory?id=fc9befe6bf6f47f89c6f34c7e855d045&srsltid=AfmBOop1DcwfJs9J_B-eIz7jfPaTNFrAKEXSfBx05SgGC2J1yujenFWD

3. Langley G, Moen R, Nolan K, Norman C, Provost L. The Improvement Guide: A Practical Approach to Enhancing Organizational Performance. 2nd ed. San Francisco: Jossey-Bass; 2009.

4. Batalden PB, Davidoff F. What is “quality improvement” and how can it transform healthcare? Quality and Safety in Health Care. 2007 Feb 1;16(1):2–3.

5. Wilcock PM, Stewart Brown GC, Bateson J, Carver J, Machin S. Using patient stories to inspire quality improvement within the NHS Modernization Agency collaborative programmes. Journal of Clinical Nursing. 2003 May;12(3):422–30.
