# Supplementary material for: Integrating theory and practice: the core components guide for rigorous quality improvement design
Source: Front Health Serv. 2026 Mar 25;6:1751580. doi: 10.3389/frhs.2026.1751580 (PMC13057544; doi:10.3389/frhs.2026.1751580)
Supplement: Supplementary file 2 [file Table2.docx]

**Supplemental File 2: System Understanding Activities**

**Problem Statement and Diagnosis**– An accurately understood and well-articulated problem statement is a key output of this Core Component. Problem diagnosis involves drawing upon the perspectives of those close to the system, as well as insights from qualitative and quantitative data. Recommended tools for supporting problem diagnosis include process maps (1), cause-effect diagrams (2), and other root-cause analysis tools (3).

**Context Assessment**– Assessing the context and environment in which a QI initiative will be implemented aids in identifying potential influencers on the project’s success. This includes factors related to the environment, social conditions, culture, organization, or the improvement team. Context assessment contributes to the tailoring of content and execution theories to mitigate potential barriers and informs evaluative measures that will contribute to understanding of project impacts. Frameworks and tools such as Practical, Robust, Implementation and Sustainability Model (4), the Consolidated Framework for Implementation Research (5), the Model for Understanding Success in Quality (6) and ATLAS (7), among others, may be used to assess context and predict success.

**Evidence Review**– A review of formal and informal evidence supports understanding of the broader landscape related to the work of the project. This includes what is known about the problem and its potential solutions as well as who has successfully addressed this problem in the past. Evidence review may include desk review, expert meetings, and site visits.

**Data Review** – Analysis of system data to understand past and current performance (often including baseline data collection), variation across identified subsystems, and populations disproportionally impacted informs the development of a measurable improvement aim for the project and contributes to the burning platform or sense of urgency for the work. Data insights may be drawn on quantitative outcome and process data, but also qualitative data on the experience of those doing the work of the system (health care workers) and those who are recipients of the system’s outputs (patients and families).

**Actor Engagement** – Development of the 6 Core Components requires understanding of and input from the different people within the system. Identification and early engagement with these different groups of actors – to understand what matters to them and assess their likely engagement in the project – is a key contributor to system understanding and sets the stage for effective co-design of the project. An expert meeting is one way to engage subject-matter experts and people with lived experience and incorporate their knowledge and perspectives into design of the 6 Core Components. When identify actors to engage or selecting content or subject matter experts, QI designers may reflect on:

- Who is (not) participating (in what ways)?
- What positionality do different team members bring and how might their perspectives affect the work? What perspectives might be missing?
- What power dynamics are at play? In what ways are people affected by the work engaged in decision-making?

Note, this guide intentionally uses the term “actor” instead of “stakeholder” as one effort to decolonize improvement practice (8).

3 Part Data Review

Advancing population health and equity while dismantling racism and other systems of oppression requires deeply understanding the experiences and realities of those in a specific population of focus (whether that is based on geography or based on some health or population feature, such as seniors experiencing food insecurity). Having this shared understanding is core to co-defining aims; building meaningful and equitable collaboration and partnerships; and designing effective, equitable, and sustainable care and service delivery systems.

**The 3-Part Data Review** draws on asset-based inquiry (rather than deficit-based thinking), to surface not only “needs” and/or “opportunities” but, importantly, to understand the ways in which systems have discarded or undervalued the assets of individuals and communities, and then to work together to ensure that all can contribute to advancing population health and well-being and dismantling inequities. This approach explores data and information (both quantitative and qualitative) from multiple perspectives– especially of those in and who work with the population of focus.

https://www.ihi.org/library/publications/population-health-guide-undertaking-three-part-data-review

**References**

1. Flores E, Ortiz DA. Process mapping as a tool for improvement. 2023 [cited 2025 Oct 31]; Available from: https://www.researchgate.net/doi/10.13140/RG.2.2.10805.14566

2. Kumah A, Nwogu CN, Issah AR, Obot E, Kanamitie DT, Sifa JS, et al. Cause-and-Effect (Fishbone) Diagram: A Tool for Generating and Organizing Quality Improvement Ideas. Global Journal on Quality and Safety in Healthcare. 2024 May 1;7(2):85–7.

3. Holifahtus Sakdiyah S, Eltivia N, Afandi A. Root Cause Analysis Using Fishbone Diagram: Company Management Decision Making. JABTER. 2022 Aug 30;1(6):566–76.

4. Feldstein AC, Glasgow RE. A practical, robust implementation and sustainability model (PRISM) for integrating research findings into practice. Jt Comm J Qual Patient Saf. 2008 Apr;34(4):228–43.

5. Damschroder LJ, Reardon CM, Opra Widerquist MA, Lowery J. Conceptualizing outcomes for use with the Consolidated Framework for Implementation Research (CFIR): the CFIR Outcomes Addendum. Implementation Science. 2022 Jan 22;17(1):7.

6. Kaplan HC, Provost LP, Froehle CM, Margolis PA. The Model for Understanding Success in Quality (MUSIQ): building a theory of context in healthcare quality improvement. BMJ Qual Saf. 2012 Jan;21(1):13–20.

7. ATLAS Initiative: Context Assessment [Internet]. Ariadne Labs. [cited 2025 May 20]. Available from: https://www.ariadnelabs.org/atlas-initiative/

8. Reed MS, Merkle BG, Cook EJ, Hafferty C, Hejnowicz AP, Holliman R, et al. Reimagining the language of engagement in a post-stakeholder world. Sustain Sci. 2024 July;19(4):1481–90.
