# Supplementary material for: Integrating theory and practice: the core components guide for rigorous quality improvement design
Source: Front Health Serv. 2026 Mar 25;6:1751580. doi: 10.3389/frhs.2026.1751580 (PMC13057544; doi:10.3389/frhs.2026.1751580)
Supplement: Supplementary file 3 [file Table3.docx]

**Supplemental File 3: Content Theory vs. Execution Theory**

| **Dimension** | **Content Theory** | **Execution Theory** |
| --- | --- | --- |
| **Primary purpose** | Explains **what** the changes are and **why** successfully implementing them should lead to the achieving the desired aim(s). | Explains **how** the work is organized to facilitate the adoption and adaptation of changes to improve short-, medium- and long-term outcomes. |
| **Core question it answers** | *What changes are we introducing, and why should they work?* | *What inputs, activities and methods, and outputs are expected to enable operationalization of the content theory in alignment with expected outcomes?* |
| **Primary focus** | The content of focus, including evidence- and practice-based interventions, and systems drivers. | Explicit process for supporting learning, testing, adapting, adopting, reporting and improving. |
| **What it specifies** | The **essential interventions** and **systems enablers** and the rationale linking them to improved outcomes. | The **methods and processes (input and outputs)**  by which teams will learn about, and receive support to test, refine, adopt, adapt and implement interventions in their unique contexts. |
| **Core Tools** | Driver Diagram and Change Package. | Logic Model, Project Roadmap and an improvement methodology (e.g. Model for Improvement, Lean, etc.). |
| **How is it Used by Project Team (**the team responsible for the initiative’s design and for supporting improvement teams ) | **Design of the Project** – to align on the specific changes/interventions and systems enablers that will be the focus of the project and the logic underpinning this.  **Implementation of the Project** – to provide a core set of changes/interventions for improvement teams to focus on.  **Learning and Evaluation of the Project** – to learn which changes/interventions are most feasible, adopted and effective , and help with assessing the causal link between the interventions of focus and improvements in process or outcome measures. | **Design of the Project** – to align on the specific inputs, outputs, activities and high-level sequencing and timeline to support improvement teams and achieve intended outcomes.  **Implementation of the Project** – To provide a preliminary plan for how to train, coach and support the improvement teams.  **Learning and Evaluation of the Project** – to help understand whether the inputs, outputs, activities and sequencing are sufficient to help the improvement teams to test, adapt and adopt the interventions of focus and achieve intended outcomes. |
| **How Used by Improvement Teams** (the teams that will test, adapt and adopt the changes at their organizations) | **Implementation of the Project –** to provide focus to their improvement work via a core set of interventions/changes and systems levers to be tested to achieve the aims of the initiative along with the content theory underpinning this. | **Implementation of the Project –** to support adhering to the activities and using the methods, tools and supports provided by the project team as they test, adapt and implement the changes/interventions of focus. |
| **Failure mode (if weak)** | Changes lack coherence or causal plausibility. | Interventions are poorly understood, tested, adapted, adopted, and/or sustained  Context/environment is not conducive to testing or adopting changes. |
