# Supplementary material for: Integrating theory and practice: the core components guide for rigorous quality improvement design
Source: Front Health Serv. 2026 Mar 25;6:1751580. doi: 10.3389/frhs.2026.1751580 (PMC13057544; doi:10.3389/frhs.2026.1751580)
Supplement: Supplementary file 4 [file Table4.docx]

**Supplemental File 4: Global Comfort Promise Case Study**

The Global Comfort Promise (GCP), a QI project led by IHI in partnership with St. Jude Children’s Research Hospital serves as a useful case study of how the 6 Core Components may be used to guide the effective design and delivery of a large-scale QI project. The content theory and measurement strategy were developed using a series of expert panels with subject matter experts and people with lived experience over a period of four months.

| Core Component | Description | Outputs |
| --- | --- | --- |
| System Understanding | Needle-based procedures are the most common source of pain and anxiety for children (1,2). Evidence-based interventions have demonstrated to be effective in reducing pain (3,4). However, these interventions have not been widely adopted globally (4). | Problem Statement  Evidence Review  Data Review  Actor Engagement  Context Assessment (using Model for Understanding Success in Quality) |
| Improvement Aim | Reduce the percentage of children with cancer who experience needle-based procedural pain in 22 participating hospitals across 18 countries by 50% from a baseline mean of 64% between July 2023 and June 2024. | Aim Statement |
| Content Theory | Evidence-based intervention: The Global Comfort Promise bundle is comprised of five evidence-based interventions:   - Topical anesthetics, - Comfort positioning, - Age-appropriate distraction techniques, - Breastfeeding or sucrose, and - Effective communication.   The Driver Diagram to promote the reliable adoption and implementation of these evidence-based bundle includes four structural, technical, or culture-based primary drivers:   - Ensure access to materials and clinical space for procedural interventions, - Adopt standardized and efficient procedural pain processes, - Improve teamwork within educated and engaged clinical workforce, and - Co-design with and actively engage patients and parents. | Driver Diagram  Change Package |
| Measurement, Evaluation, and Learning | The GCP measurement strategy includes a family of measures (outcome, process and balancing). To guide learning about the project overall and its effectiveness, the GCP Project team developed an Evaluation and Learning plan using a mixed methods approach and a quasi-experimental design. | Measurement Strategy (including measurement guide, data collection forms, and platform for reporting and visualizing data via run charts)  Evaluation and Learning Plan |
| Execution Theory | The GCP Project used a modified Breakthrough Series Model (5) design, including required monthly coaching and optional monthly office hours for improvement teams. Key inputs, outputs, and outcomes were outlined using a logic model, with projections on expected progress throughout the project period included in a roadmap. | Logic Model  Project Roadmap |
| Dissemination and Communication | GCP developed a multi-modal dissemination, sharing, and communications plan that included lay and professional audiences through blogs, how-to guides, workshops, conference presentations, and peer-reviewed publications. | Communication Plan  Dissemination Strategy |

**References**

1. Walther‐Larsen S, Pedersen MT, Friis SM, Aagaard GB, Rømsing J, Jeppesen EM, et al. Pain prevalence in hospitalized children: a prospective cross‐sectional survey in four Danish university hospitals. Acta Anaesthesiol Scand. 2017 Mar;61(3):328–37.

2. Friedrichsdorf SJ, Postier A, Eull D, Weidner C, Foster L, Gilbert M, et al. Pain Outcomes in a US Children’s Hospital: A Prospective Cross-Sectional Survey. Hosp Pediatr. 2015 Jan;5(1):18–26.

3. Friedrichsdorf SJ, Eull D, Weidner C, Postier A. A hospital-wide initiative to eliminate or reduce needle pain in children using lean methodology. Pain Rep. 2018 Sept 11;3(Suppl 1):e671.

4. McNeil MJ, Garcia Quintero X, Gonzalez M, Zheng Y, Ugaz Olivares C, Morales R, et al. Preventing and Treating Pain and Anxiety during Needle-Based Procedures in Children with Cancer in Low- and Middle-Income Countries. Cancers (Basel). 2024 Mar 1;16(5):1025.
